# Event and Comment

**Published:** 1923-02

**Authors:** 


### DENTAL REGISTER

THE MAGAZINE OF DENTISTRY

Vol. LXXVII FEBRUARY, 1923 No. 2

### EVENT AND COMMENT

BY VOTRE AMI DENTAIRE

After hearing Dr. Coué in Cincinnati one is impressed with the sincerity of the gentleman and the candor with which he enunciates his theory of Auto-Suggestion. The summation of the principle could be stated as meaning that, "what is physically possible can be done by one who has confidence in himself or herself to do it." Even if it is possible to do it, the Auto-Suggestion in one's mind that it can not be done, governs, and it is not attempted. Imagination rules.

As for his formula, "every day, etc.," it is designed as a tonic. The "every way" expression meaning "general," therefore a general tonic including all parts of the body. The rapid repetition of the phrase is to prevent the contrary suggestion to have any chance to effect a contrary suggestion while the phrase suggestion is being repeated.

---

Safety is one of the slogans in all kinds of service and can be applied to X-ray work by the use of the Thwaites X-ray Machine. The safety of this outfit is obtained by eliminating the ground wire which in other machines is supposed to be a safety device. In the Thwaites, however, the elimination of the ground wire makes it safe. There is no static in the machine from the induction coil, therefore no charge of electricity seeking the ground through adjacent objects. The Thwaites X-ray outfit was for several years handled by a single firm for the general distribution in the United States, but now is furnished by dental dealers all over the United States. It is of interest to know that safety in X-rays can be attained by use of the Thwaites machine.

---

It is intended that the coming meeting of the Kentucky State Dental Association shall be an unusually interesting one. The committees are working hard to accomplish this and to make the 1923 meeting the largest and most interesting meeting yet. Following is the preliminary program:

Boyd S. Gardner of Mayo Clinic.

C. Bruce Clark of Uniontown, Pa., "The Fixed Bridge."

Bowen K. Bowen of Nashville, Tenn., "Lower Dentures."

Harold O. Brown, Rochester, New York, "Theory and Practice of Partial Dentures."

Seven Kentucky Clinicians, 45-minutes each.

Entertainments for the ladies—Golf tournament and dance.

Visiting dentists welcome.

C. E. Hume, President, Francis Bldg., Louisville, Ky.

R. L. Sprau, Chairman Executive Committee, Baxter Ave., Louisville, Ky.

